# (±)-5-bromo-2-(5-fluoro-1-hydroxyamyl) Benzoate Protects Against Oxidative Stress Injury in PC12 Cells Exposed to H_2_O_2_ Through Activation of Nrf2 Pathway

**DOI:** 10.3389/fphar.2022.943111

**Published:** 2022-07-18

**Authors:** Saidan Qi, Xiaojiao Zhang, Zhenzhen Fu, Anran Pi, Feiyan Shi, Yanan Fan, Jiahua Zhang, Tingting Xiao, Dong Shang, Meng Lin, Na Gao, Junbiao Chang, Yuan Gao

**Affiliations:** ^1^ Department of Pharmacology, School of Basic Medicine, Zhengzhou University, Zhengzhou, China; ^2^ Department of Experimental Center, School of Medicine, Zhengzhou University, Zhengzhou, China; ^3^ Department of Institute of Clinical Pharmacology, Zhengzhou University, Zhengzhou, China; ^4^ Department of Chemistry and Molecular Engineering, Zhengzhou University, Zhengzhou, China

**Keywords:** BFB, H_2_O_2_, PC12 cell, Nrf2, autophagy

## Abstract

**Background:** Oxidative stress is associated with the pathogenesis of ischemic stroke (±)-5-bromo-2-(5-fluoro-1-hydroxyamyl) benzoate (BFB) is a novel compound modified by dl-3-n-butylphthalide (NBP). Here, we hypothesized that BFB may protect the PC12 cells against H_2_O_2_-induced oxidative stress injury through activation of the Nrf2 pathway.

**Methods:** We measured the cell viability and levels of lactate dehydrogenase (LDH), malondialdehyde (MDA), glutathione (GSH), and reactive oxygen species (ROS) to determine the construction of the H_2_O_2_-induced models of oxidative stress in PC12 cells. Additionally, apoptotic cell death, mitochondrial membrane potential, and cellular morphology were examined to determine the effect of BFB on oxidative stress injury in H_2_O_2_-treated PC12 cells. The expression levels of Nrf2-related and autophagy-related genes and proteins were detected using real time quantative PCR (RT-qPCR), Western Blot, and immunofluorescence analyses.

**Results:** Our study showed that BFB treatment reduced the elevated levels of MDA, LDH, and ROS, and decreased cell viability and GSH in H_2_O_2_-treated PC12 cells. We also observed the elevated expression of Nrf2 pathway-related factors and intranuclear transitions and found that Nrf2 inhibitors (ML385) could block the protective effect of BFB. The inhibitory effect of BFB on oxidative stress may be partially regulated by Nrf2 activation, and the initiation and induction of autophagy.

**Conclusion:** BFB inhibited H_2_O_2_-induced oxidative stress injury in PC12 cells by activating the Nrf2 pathway, initiating and inducing autophagy, suggesting that BFB may be a promising therapeutic agent in treating neurological disorders like cerebral ischemia.

## 1 Introduction

Epidemiological studies have revealed that about 15 million people worldwide suffer from stroke every year, and the World Health Organization has listed stroke as an upcoming epidemic of the 21st century (Thrift et al., 2017). The disease causes severe physical damage and has a poor prognosis (Bennion et al., 2018). At present, no treatment is entirely successful (Bennion et al., 2018). Stroke can be roughly divided into two types: ischemic and hemorrhagic stroke (Austin et al., 2016). Ischemic stroke is one of the major neurological disorders globally, with high rates of mortality, disability, incidence, and recurrence. Ischemic stroke occurs when the blood supply to the brain is reduced, usually due to thromboembolism or atherosclerotic occlusion which results in a lack of supply of oxygen and nutrients to the brain (Heiss, 2010). Oxidative stress mediates the occurrence and development of ischemic stroke, resulting in excessive reactive oxygen species (ROS) production (Woodruff et al., 2011). ROS, a series of oxygen intermediates, leads to lipid peroxidation damage in the brain tissues (Xiang et al., 2021). Some endogenous enzymes and non-enzymes such as superoxide dismutase (SOD) and glutathione (GSH) can be involved in tissue repair after ischemia-reperfusion injury (Margaill et al., 2005; Zhou et al., 2016). These enzymes are encoded by specific genes that carry antioxidant response elements in promoters (Zhang et al., 2015). Nuclear factor erythroid factor 2-related factor (Nrf2) plays a vital role in regulating the activation of these genes (Shirley et al., 2014).

The Nrf2 pathway is one of the most important pathways in inhibiting oxidative stress (Zhang et al., 2021). Ubiquitin-binding autophagy receptor protein p62/SQSTM1 (p62) is an upstream regulator of the Nrf2 pathway, and acts as a bridge between the Nrf2 pathway and autophagy (Frias et al., 2020). When ROS stimulates cells, p62 phosphorylation aggregates LC3 and degrades Keap1, increasing its affinity to Keap1 and forming a p62-Keap1 dimer (Zhang et al., 2021). To upregulate the Nrf2 pathway, Nrf2 is transferred into the nucleus to activate downstream antioxidant genes such as HO-1 and SOD. Interestingly, Nrf2 can also stimulate autophagy. As a common self-repair mechanism in eukaryotic cells, autophagy regulates cellular growth, metabolic homeostasis, and cellular defense against oxidative stress (Dikic, 2017). When many Nrf2s translocate to the nucleus, the transcription of autophagy-related genes, including Atg5, p62, and LC3B, is upregulated by binding to ARE. Reports showed that clinical applications are limited due to a narrow therapeutic window (no more than 6 h) and adverse reactions (Hurd et al., 2021).

Dl-3-n-butylphthalide (NBP) was approved by China Food and Drug Administration in 2002 to treat ischemic stroke (Peng et al., 2004). However, the treatment is associated with adverse reactions such as elevated transaminase, gastrointestinal discomfort, and allergy (Marco-Contelles and Zhang, 2020) (±)-5-bromo-2-(5-fluoro-1-hydroxyamyl) benzoate (BFB) is a new compound based on NBP substituted with halogens. We found that BFB possesses antioxidant, anti-inflammatory, and neuroprotective properties in MCAO-induced focal cerebral ischemia in rats (data not published). In this study, we hypothesized that the neuroprotective effects of BFB are based on the suppression of neuronal apoptosis by activating the Nrf2 signaling pathway in H_2_O_2_-induced oxidative stress injury in PC12 cells.

## 2 Materials and Methods

### 2.1 Reagents and Antibodies

(±)-5-bromo-2-(5-fluoro-1-hydroxyacyl) benzoate (BFB) (purity>98%) was brought from Henan Genuine Biotech Co., Ltd. (Henan, China). dl-3-n-butylphthalide (NBP) was purchased from National Institutes for Food and Drug Administration (Beijing, China). BFB and NBP were dissolved as a stock solution in dimethylsulfoxide (DMSO) and diluted with cell culture medium before the experiment. H_2_O_2_ was purchased from Sigma-Aldrich Co., Ltd. (St. Louis, Missouri, United States). ML385 (846,557-71-9) was purchased from AbMole Co., Ltd. (Houston, Texas, United States). All cell culture medium components, dimethyl sulfoxide (DMSO) (D8371), BCA protein analysis kit (PC0020) and CCK8 (PC0020) Assay Kit were brought from Solarbio Co., Ltd. (Beijing, China). DCFH-DA Probe Kit (KGAF018) and Annexin V-FITC/PC kit (KGA105-KGA108) were brought from keygen biotech Co., Ltd. (Jiangsu, China). LDH (A020-2), MDA (A001-3-1) and GSH (A006-2-1) biochemical kits were brought from Nanjing Jiancheng Co., Ltd. (Jiangsu, China). Rh123 (HY-D0816) was purchased from MedChemExpress Co., Ltd. (Monmouth Junction, NJ). In this study, the following primary antibodies were used for Western blot (WB) or immunofluorescence (IF) analysis: Bax (ab32503, Abcam, 1:5,000 for WB),Bcl-2 (ab182858, Abcam, 1:2000 for WB), caspase-3 (ab184787, Abcam, 1:2000 for WB), p62 (66184-1-Ig, Proteintech, 1:5,000 for WB), Keap1 (10503-2-AP, Proteintech, 1:2000 for WB), Nrf2 (AF0639, Affinity Biosciences, 1:1,000 for WB), HO-1 (10701-1-AO, Proteintech, 1:1,000 for WB), LC3 (4,599, Cell Signaling Technology, 1:1,000 for WB), Beclin-1 (ab207612, Abcam, 1:2000 for WB), Beta Actin (66009-1-Ig, Proteintech, 1:2000 for WB), Histone H3 (AF0863, Affinity Biosciences, 1:1,000 for WB). Peroxidase-conjugated second antibody (HA1006, HA1001) was brought from HuaBio Biotechnology Co., Ltd. (Zhejiang, China).

### 2.2 Cell Culture

This work acquired the differentiated PC12 cells in Cell Resource Center of Shanghai Institute of Biological Science, Chinese Academy of Science (Shanghai, China). Briefly, we cultured PC12 cells within the DMEM that contained 10% fetal bovine serum (FBS), then nurtured under 37°C and 5% CO_2_/95% conditions within the humid cell incubator.

This work later classified PC12 cells as six groups below: Control, H_2_O_2_, NBP, BFB 1, 15, 30 µm groups. BFB at diverse doses was utilized to precondition PC12 cells for a 3-h period, whereas Control cells received 0.9% saline instead, followed by 400 µm H_2_O_2_ treatment of PC12 cells for a 24-h period.

In addition, the cells were classified into six groups, respectively, Control, H_2_O_2_, NBP (30 μmol/L), Nrf2 inhibitor ML385 (5 µm), BFB (30 µm), as well as ML385 + BFB groups. This work incubated PC12 cells for a 0.5-h period using 5 μm ML385, followed by 3-h incubation using 30 µm NBP or BFB, finally, 24-h exposure to H_2_O_2_ (400 µm).

### 2.3 Cell Viability Assays

In brief, this work inoculated PC12 cells (1 × 10^4^/well) into the 96-well plates, followed by overnight culture to make cells adhere to the wall with appropriate density. BFB at diverse doses was then added to treat PC12 cells at 37°C for a 3-h period before exposure to H_2_O_2_. Thereafter, we added freshly prepared medium (100 μl) that contained CCK-8 solution (10 μl) for replacing the original medium, followed by 0.5–2-h incubation under 37°C after washing by PBS. Absorbance (A values) were determined with the microplate reader ELX800 (Bio-Tek, Norcross, GA, United States) at 450 nm.

### 2.4 ROS Assay

DCFH-DA penetrating cell membrane was subject to hydrolysis into membrane-impermeable DCFH, which accumulated inside the cell and was further oxidized by ROS to DCF with fluorescence, thus reflecting the ROS level. This work incubated PC12 cells (1 × 10^4^/well) into 96-well plates, followed by digestion using EDTA-free trypsin at the end of treatment. The DCFH-DA fluorescent probe was diluted to the desired amount at a ratio of 1:1,000 before the experiment. 10 μm DCFH-DA was added to incubate cells under 37°C for a 20-min period. After washing thrice, flow cytometer was utilized to measure fluorescence intensity in 480 nm excitation and 525 nm emission. Meanwhile, Nikon ECLIPSE Ti2 fluorescence microscopy (Tokyo, JAPA) was used to observe fluorescent intensities.

### 2.5 Measurement of LDH-Leakage, MDA and GSH Contents

Samples were centrifuged for a 5-min period at 1,380 x g and 4°C to collect cells. After rinsing thrice by PBS, cells were added into the lysis buffer including 20 mm Tris (pH 7.5), 150 mm NaCl, 1 mm PMSF, 1% Triton X-100 for homogenization, and cell supernatants were collected. The LDH-cytotoxicity assay kit was used to analyze LDH leakage. GSH and MDA contents were measured by adopting commercially available kits.

### 2.6 Apoptosis Rate Determination

Apoptosis was measured through flow cytometry (FCM). In brief, BFB or NBP at diverse doses was applied in pretreating PC12 cells, and cells were then exposed to H_2_O_2_. After collection, cells were rinsed by PBS twice. Afterwards, FITC-Annexin V/propidium iodide (PI) was utilized to stain cells with the Annexin V-FITC Apoptosis Detection Kit, and detected using Agilent NovoCyte 2060R (San Diego, CA, United States).

### 2.7 MMP and Mitochondrial Ultrastructure Observation

Changes of MMP were determined through fluorescence microscopy or fluorescence microplate reader with Rh123 fluorescent dye according to previous description. In brief, after resuspension within PBS, cells were subject to 30-min incubation using 10 mm Rh123 under 37°C, and washing twice by 0.1 M PBS (pH 7.4). Fluorescence microplate reader was then employed to quantify fluorescence intensity of cells at the emission and excitation wavelengths of 525 and 488 nm, respectively.

This work utilized transmission electron microscopy (TEM) for observing the ultrastructure of mitochondria. In brief, following specific treatment, 2.5% glutaraldehyde was added to fix cells for a 2-h period in a cell culture medium, followed by 30-min post-fixation using 1% osmium tetroxide. Thereafter, gradient ethanol contents were added to dehydrate cells for a 15-min period each, followed by embedding. Samples were cut, while the Hitachi HT7700 transmission electron microscope (Tokyo, Japan) was employed for analysis.

### 2.8 Protein Extraction and Western Blot

After treatment, pre-chilled PBS was added to rinse PC12 cells, and a scratch was made in the dishes containing 1 ml PBS, followed by 10-min centrifugation of cell homogenates at 1,500 rpm. Afterwards, the Beyotime RIPA cell lysis buffer was adopted for extracting cellular proteins using 1 mm PMSF in line with specific protocols. Additionally, we then separated nuclear and cytoplasmic protein fractions in line with instructions of Beyotime nuclear and cytoplasmic extraction kit. The Beyotime BCA protein assay kit was employed for measuring protein content. Thereafter, each sample was preserved under –80°C in WB assay. Protein separation was conducted through 10% SDS-PAGE, and later transferred onto the 0.45-μm PVDF membranes. After 2-h blocking under ambient temperature, membranes were subject to 14-h incubation using primary antibody under 4°C. The next day, the excess primary antibody was eluted using TBST, followed by 2-h secondary antibody incubation under ambient temperature, and finally treated by ECL chemiluminescent substrate for observation and grayscale analysis with ImageJ software.

### 2.9 RNA Extraction and RT-qPCR Analysis

This work inoculated PC12 cells (2 × 10^5^/well) into the 6-well plates, then lysed at room temperature using RNA extraction reagent (TrizoL) and RNA was extracted. Finally, the concentration of each group of RNA was measured in Thermofisher Nanodrop One (Waltham, Massachusetts, United States) and recorded. This work later collected total RNA (1 μg) to prepare cDNA using a reverse transcription kit (RR047A, Takara, Japan). SYBR qPCR Master Mix kit was later used to assay mRNA levels on QuantStudioTM six Flex Real-Time PCR System (Singapore). Sequences of PCR primers used were presented below.

GAPDH: Forward 5′ -AAG​TTC​AAC​GGC​ACA​GTC​AA-3′

Reverse 5′ -GAT​CTC​GCT​CCT​GGA​AGA​TG-3′;

p62: Forward 5′ -GTG​GTC​GTG​GGG​TGT​CTG​T-3′,

Reverse 5′ -GGA​GCC​TCT​TAC​TGG​GGT​TC-3′;

Keap1: Forward 5′ -ACA​TCT​ACG​CAG​TCG​GGG​G-3′, Reverse 5′ -TAC​AGC​AAG​CGG​TTG​AGC​AC-3′;

Nrf2: Forward 5′ -CAT​TCA​AGC​CGA​TTA​GAG​G-3′, Reverse 5′ -TTG​CTC​CTT​GGA​CAT​CAT​TT-3′;

HO-1: Forward 5′ -GCC​TGG​CTT​TTT​TCA​CCT​T-3′, Reverse 5′ -TGT​TCA​TGC​GAG​CAC​GAT​A-3′;

SOD1: Forward 5′ -GAG​ACC​TGG​GCA​ATG​TGG-3′, Reverse 5′ -AAG​TCA​TCT​TGT​TTC​TCG​TGG​A-3′;

CAT: Forward 5′ -TGT​GGT​TTT​CAC​CGA​CGA-3′, Reverse 5′ -CAC​CTT​TGC​CTT​GGA​GTA​TCT-3′;

Atg4: Forward 5′ -TGC​TTT​ATC​CCC​GAC​GAG​AG-3′, Reverse 5′ -TTT​GAC​TTG​CTG​GCA​CCA​GT-3′;

Atg5: Forward 5′ -ATT​TGC​TTT​TGC​CAA​GAG​TC-3′, Reverse 5′ -GAT​AAT​GCC​ATT​TCA​GGG​GT-3′;

Rab5: Forward 5′ -TAG​CAC​CAA​TGT​ACT​ACC​GA-3′, Reverse 5′ -CTT​GCC​TTT​GAA​GTT​CTT​TA-3′.

### 2.10 Statistical Treatment

Each experiment was carried out in triplicate thrice. This work adopted one-way ANOVA for comparing Mean ± standard deviation between different groups, along with Dunnett’s post hoc test with Graphpad Prism 8.0 software. *p* < 0.05 stood for significant difference.

## 3 Results

### 3.1 H_2_O_2_ Model Establishment and BFB Mitigated the H_2_O_2_-Treated PC12 Cell Viability Loss

**FIGURE 1:**
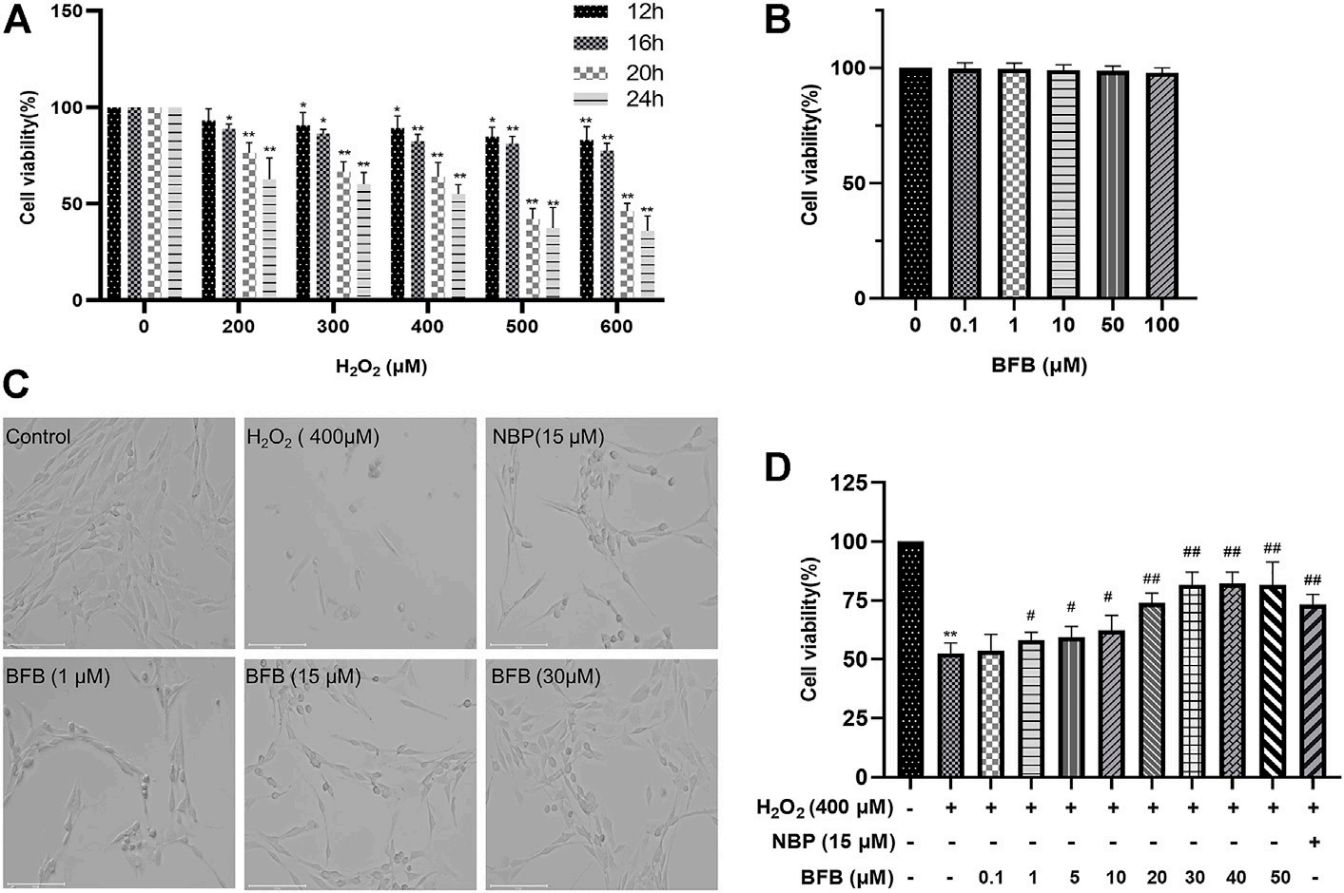
BFB’s role in H_2_O_2_-induced PC12 cell viability **(A)** H_2_O_2_ at different doses (0, 200, 300, 400, 500, 600 μm) was added to treat PC12 cells for a 24-h period, then CCK-8 assays were conducted to assess cell viability **(B)** BFB (0.1, 1, 10, 50, 100 μm)’s cytotoxicity to PC12 cells was analyzed by CCK-8 assays for a 24-h period **(C)** Bright-field images revealed H_2_O_2_-induced cell loss and alterations in cell morphology, which were remarkably abolished by administering BFB (scale bar = 125 μm) **(D)** Concentration-response curve for H_2_O_2_-induced PC12 cell viability. 400 μm H_2_O_2_ and low-glucose DMEM were added to treat PC12 cells for a 24-h period, with pretreatment with BFB (0.1–50 μm) or NBP (15 μm) for 3 h respectively. Data were analyzed by mean ± SD from 3 separate assays. *p < 0.05, **p < 0.01, versus control, ^#^ *p* < 0.05, ^##^ *p* < 0.01, versus H_2_O_2_ group.

For establishing a H_2_O_2_ model based on PC12 cells, this work adopted H_2_O_2_ to induce death of PC12 cells. CCK-8 assays were conducted to determine the best H_2_O_2_ content to induce reduction of 40–60% in PC12 cell viability. As shown in Figure 1A 400 μm H_2_O_2_ induction for a 24-h period contributed to simulating oxidative stress-mediated PC12 cell damage. BFB at increasing contents was added to treat PC12 cells for a 24-h period, while BFB at ≤50 μm did not show obvious cytotoxicity to PC12 cells relative to Control (*p* > 0.05) (Figure 1B).

Besides, BFB reversed H_2_O_2_-mediated PC12 cell viability reduction (Figures 1C,D). Compared with Control group, 24-h treatment with 400 μm H_2_O_2_ changed cell morphology, which included the reduced cell quantity markedly, membrane blebbing as well as cell shrinkage. BFB or 15 μm NBP pretreatment alleviated the above cell injuries. Meanwhile, after 3-h pretreatment of cells using BFB (0.1–50 μm), PC12 cells had significantly elevated viability dose-dependently (53.65–81.59%). BFB exhibited strong protection on PC12, and the EC_50_ was 14.74 μm. Combined with our CCK-8 assay results, BFB at 1, 15, and 30 μm was chosen in subsequent analyses.

### 3.2 BFB Attenuated H_2_O_2_-Induced ROS Generation and Lipid Peroxidation

**FIGURE 2:**
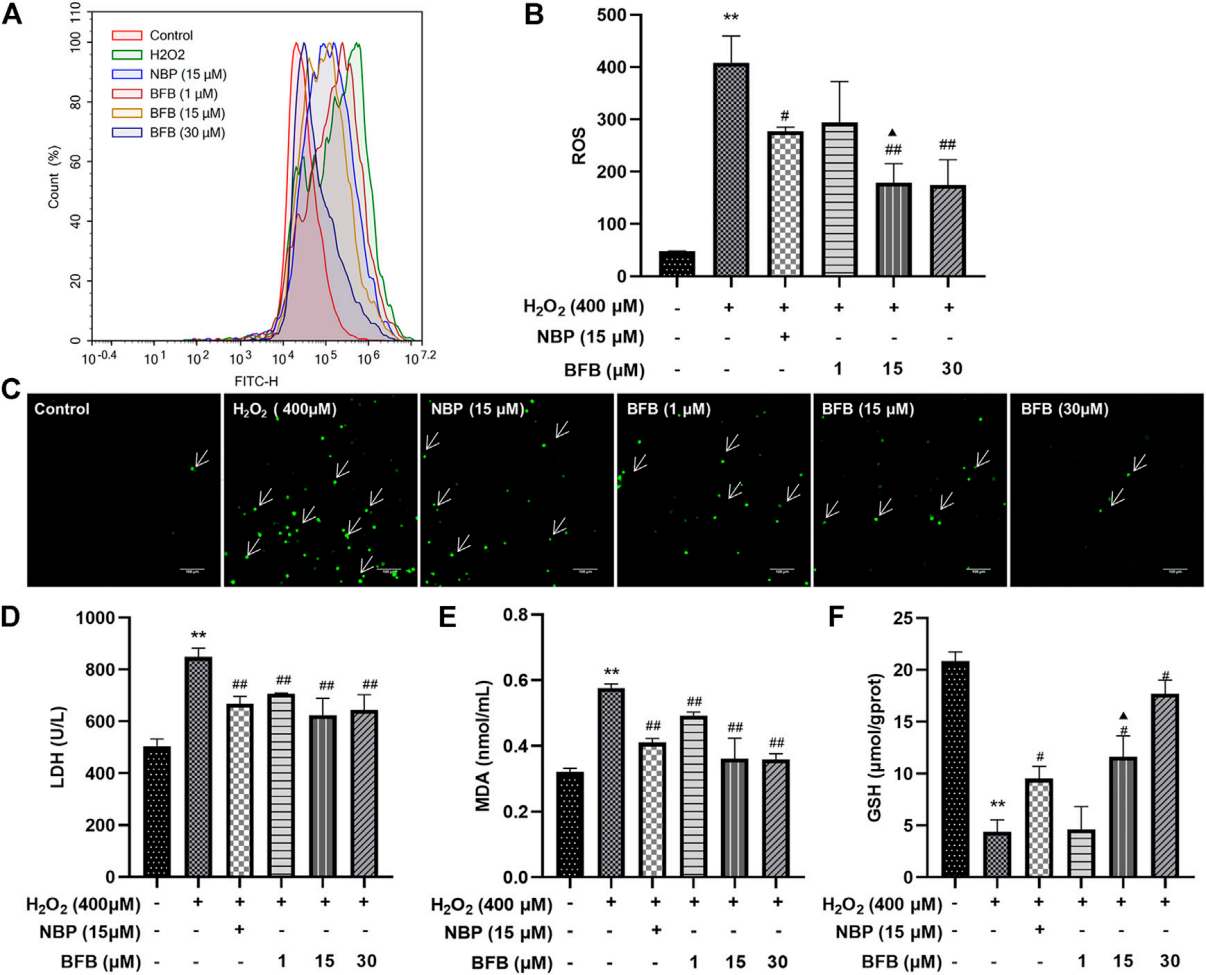
BFB alleviated OS characteristics in H_2_O_2_-exposed PC12 cells. H_2_O_2_ (400 μm) was added to treat PC12 cells for a 24-h period with elevating concentrations of BFB (1, 15, 30 μm) or NBP (15 μm) for a 3-h period, followed by measurement of oxidative stress-associated indicators **(A,B)** ROS generation analyzed by DCFH-DA through FCM **(C)** Fluorescence microscopy images of the protective effects of BFB against H_2_O_2_. Administration of 400 µm H_2_O_2_ significantly increased ROS accumulation relative to Control. BFB significantly decreased ROS compared with 400 µm H_2_O_2_ group, and the reduction in ROS expression increased with the BFB concentration. Cell GSH **(D)** LDH leakage **(E)** MDA **(F)** GSH contents were determined using commercially available kits. Data were analyzed by mean ± SD from 3 separate assays. *p < 0.05, **p < 0.01, versus control, ^#^ *p* < 0.05, ^##^ *p* < 0.01, versus H_2_O_2_ group. ^▲^ *p* < 0.05, ^▲▲^ *p* < 0.01, versus NBP group.

As shown in Figure 2A–C 24-h treatment with 400 μm H_2_O_2_ remarkably elevated fluorescence intensity in cells (407.79 ± 51.71; *p* < 0.01) relative to Control (47.69 ± 0.64). Following pretreatment with BFB (15, 30 μm), the fluorescence intensity decreased significantly (179.14 ± 36.06, 174.39 ± 48.22; *p* < 0.01). 15 µm BFB group was superior to the equimolar concentration of the NBP group (276.96 ± 8.13; *p* < 0.05). As a result, H_2_O_2_ increased ROS production which attenuated with BFB.

As shown in Figures 2D–F, cellular LDH release remarkably elevated of H_2_O_2_ group (848.94 ± 33.82 U/L; *p* < 0.01) relative to Control (505.11 ± 27.27 U/L), however, pretreatment with 1, 15 and 30 µm BFB significantly reduced LDH (706.50 ± 2.74, 624.98 ± 63.93, 643.97 ± 58.77 U/L separately; *p* < 0.05, *p* < 0.01) versus H_2_O_2_ group leakage. MDA content of H_2_O_2_ group significantly increased (0.58 ± 0.01 nmol/ml; *p* < 0.01) relative to Control (0.32 ± 0.01 nmol/ml). By contrast, after combined treatment of PC12 cells by 400 µM H_2_O_2_ and BFB (1, 15 and 30 µm), MDA level was reduced significantly (0.49 ± 0.01, 0.36 ± 0.06, 0.36 ± 0.02 nmol/ml separately, *p* < 0.05, *p* < 0.01). GSH level showed a decreasing trend in cells exposed to 400 µm H_2_O_2_ (4.54 ± 1.58 µmol/gprot; *p* < 0.01) relative to Control (20.41 ± 0.75 µmol/gprot), whereas treatment with BFB (15 and 30 µm) remarkably mitigated GSH levels within 400 µm H_2_O_2_-treated cells (12.78 ± 0.34, 17.14 ± 1.3 µmol/gprot respectively, *p* < 0.05, *p* < 0.01).

### 3.3 BFB Showed Protection on H_2_O_2_-Mediated Apoptosis of PC12 Cells and Ameliorated Mitochondrial Dysfunction

**FIGURE 3:**
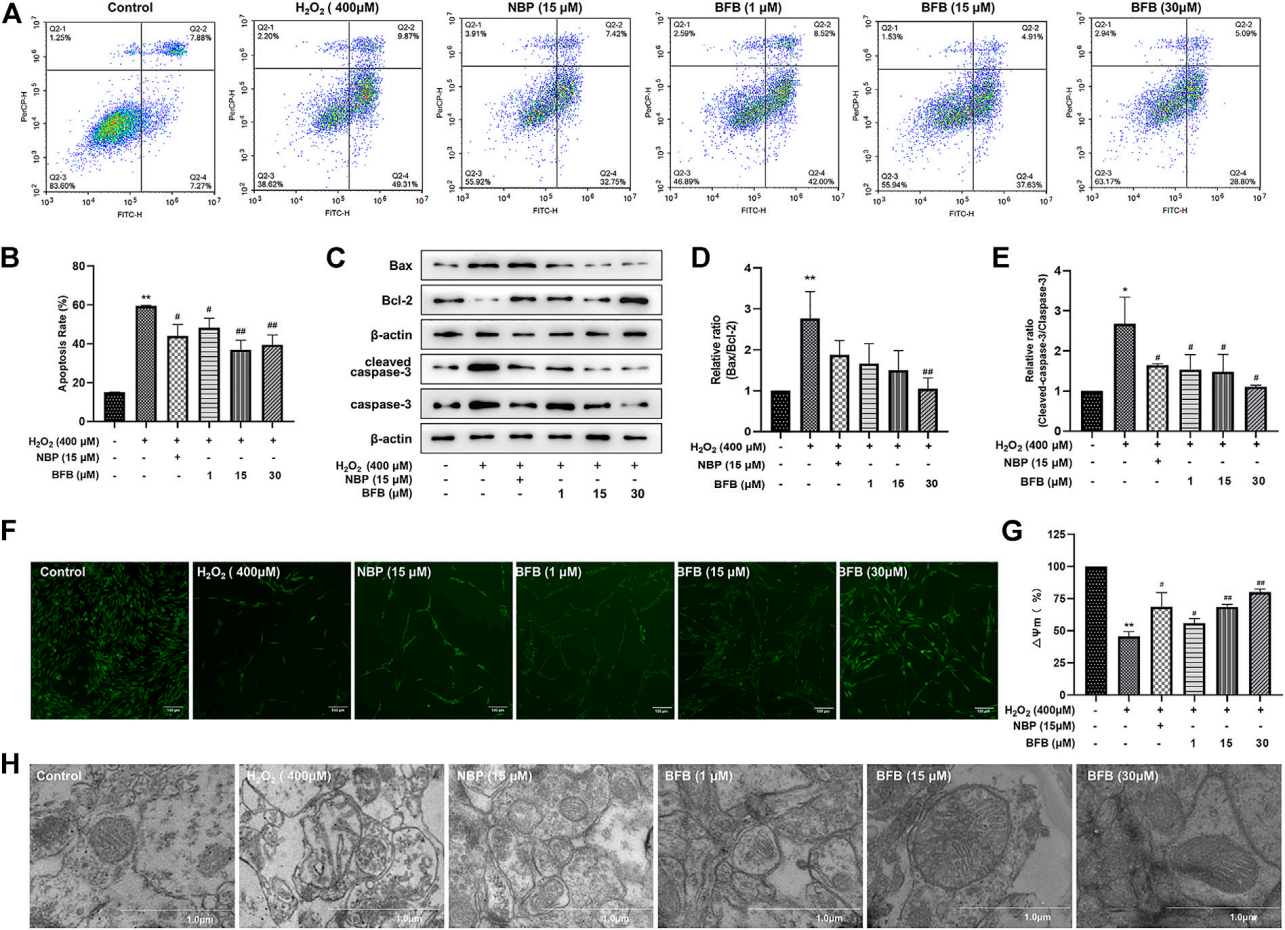
BFB inhibited apoptosis and abolished mitochondrial alterations of H_2_O_2_-induced PC12 cells **(A,B)** FCM on apoptotic rate using Annexin V/PI staining **(C)** WB assay regarding Bax, Bcl-2, capase-3, cleaved-capase three expression within PC12 cells exposed to 400 µm H_2_O_2_ and 1, 15, 30 µm BFB or NBP (15 μm). Bcl-2 protein expression was significantly upregulated and Bax, capase-3 and cleave-capase 3 decreased within BFB-treated cells relative to H_2_O_2_-treated cells **(D,E)** WB assay on Bax/Bcl-2 and cleaved-capase 3/capase-3 ratio. Ratio of H_2_O_2_ group increased compared with Control group, but BFB partially recovered this expression ratio from upregulation **(F)** Typical images displaying mitochondrial staining under diverse conditions by using fluorescence microscopy (scale bar = 100 µm) **(G)** Bar graph presenting fluorescence intensity, which indicated MMP, by using fluorescence microplate reader **(H)** Typical images showing ultrastructure of mitochondria through TEM (scale bar = 1.0 µm). Arrows stand for mitochondria. The data were represented by mean ± SD of three separate assays. *p < 0.05, **p < 0.01, versus control, ^#^ *p* < 0.05, ^##^ *p* < 0.01, versus H_2_O_2_ group. ^▲^ *p* < 0.05, ^▲▲^ *p* < 0.01, versus NBP group.

To quantitatively demonstrate BFB’s impact on H_2_O_2_-mediated apoptosis, FCM was conducted by Annexin V/PI staining. According to Figures 3A,B, for Control group, the apoptotic rate was 15.17 ± 0.04% of total cells. But, incubating cells with 400 µm H_2_O_2_ alone significantly increased apoptotic rate to 59.49 ± 0.33%. Pretreatment with BFB (1, 15 and 30 µm) markedly attenuated cell apoptosis induced by H_2_O_2_ to 48.26 ± 4.92%, 36.91 ± 4.97%, 39.57 ± 4.99%, respectively.

To observe whether BFB’s protection was mediated by suppressing apoptosis, this work analyzed Bax, Bcl-2, caspase-3 as well as cleaved caspase-3 expression within H_2_O_2_ or BFB-treated PC12 cells. After WB assay, protein levels were compared with corresponding Controls. As a result, the Bax/Bcl-2 ratio of H_2_O_2_ group significantly elevated (*p <* 0.01) versus Control. In comparison, BFB pretreatment remarkably decreased Bax/Bcl-2 ratio of the H_2_O_2_-treated cells relative to cells that were only exposed to H_2_O_2_ (*p <* 0.05, respectively). In addition, cleaved caspase-3/caspase-3 ratio of H_2_O_2_ group markedly elevated (*p* < 0.01) relative to Control, whereas administration of 1, 15, and 30 µm BFB reduced cleaved caspase-3/caspase-3 ratio of H_2_O_2_-treated cells when compared with the cells that were only exposed to H_2_O_2_ (*p* < 0.05, separately). (Figures 3C–E).

MMP distribution was considered to be the major early phenomenon in the apoptotic process. We assessed the effect of on the MMP by testing the cellular retention of Rh123 by fluorescence microscopy or fluorescence microplate reader and the observation of mitochondrial ultrastructure by transmission electron microscopy in this study. As shown in Figures 3F–H, MMP contents markedly declined to 45.48 ± 3.92% of the Control following 24-h incubation using 400 µm H2O2 in PC12 cells, whereas administration with BFB at 1, 15, 30 µm remarkably reduced the H2O2-mediated alterations of MMP. BFB elevated MMP levels (55.97 ± 3.55, 68.63 ± 1.97, 80.18 ± 2.33% separately; *p* < 0.05, *p* < 0.01), versus H2O_2_-treated PC12 cells, indicating the role of BFB in protecting against H2O2-mediated reduction in MMP of PC12 cells.

As shown in Figure 3I, the enlarged and broken mitochondria, blurred contours, mitochondrial shrinkage and reduced or loss of mitochondrial cristae within H_2_O_2_-exposed PC12 cells, along with markedly improved mitochondrial morphology was seen following BFB treatment by using transmission electron microscope.

### 3.4 BFB Regulates Nrf2 Signaling Pathway to Modulate the Levels of Pro- and Antioxidative Factors in H_2_O_2_-Induced PC12 Cells Injury

**FIGURE 4:**
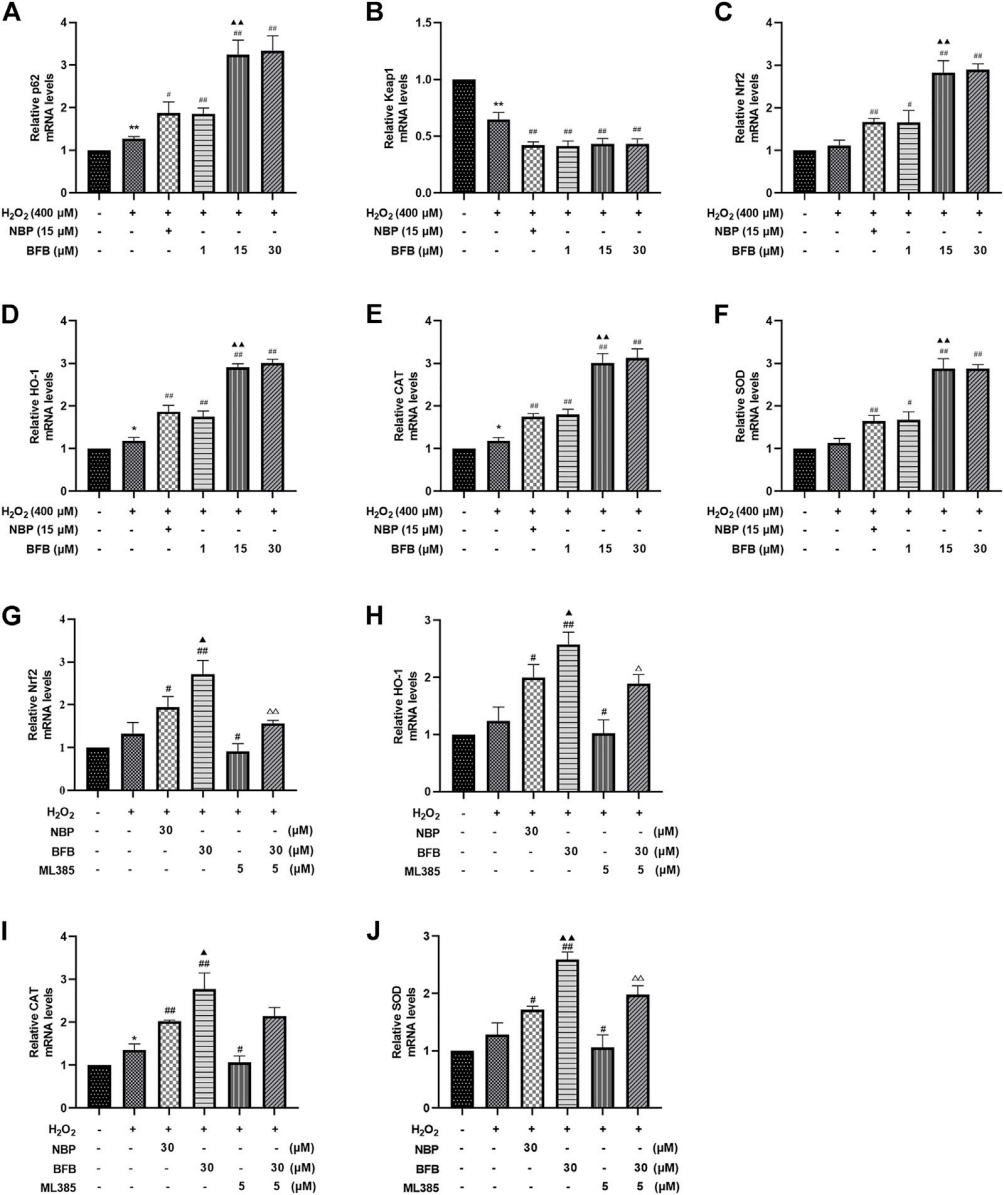
BFB’s role in cellular P62-Keap1-Nrf2 pathway-related factors expression level. H_2_O_2_ (400 μm) was added to treat PC12 cells with/without BFB (1, 5, 10 μm) or NBP (15 μm) for a 3-h period. RT-qPCR was conducted to analyze mRNA expression **(A)** p62 **(B)** keap1 **(C)** Nrf2 **(D)** HO-1 **(E)** CAT **(F)** SOD. Nrf2 suppression alleviated BFB’s inhibition against oxidative stress. 5 μm ML385 was added to pretreat PC12 cells for a 0.5-h period, followed by H_2_O_2_ (400 μm) stimulation with/without BFB (30 μm) or NBP (30 μm) **(G)**Nrf2 **(H)** HO-1**(I)** CAT **(J)** SOD. Data were represented by mean ± SD from 3 separate assays. *p < 0.05, **p < 0.01, versus Control, ^#^ *p* < 0.05, ^##^ *p* < 0.01, versus H_2_O_2_ group. ^▲^ *p* < 0.05, ^▲▲^ *p* < 0.01, versus NBP group. ^△^ *p* < 0.05, ^△△^ *p* < 0.01, versus ML385 group.

**FIGURE 5:**
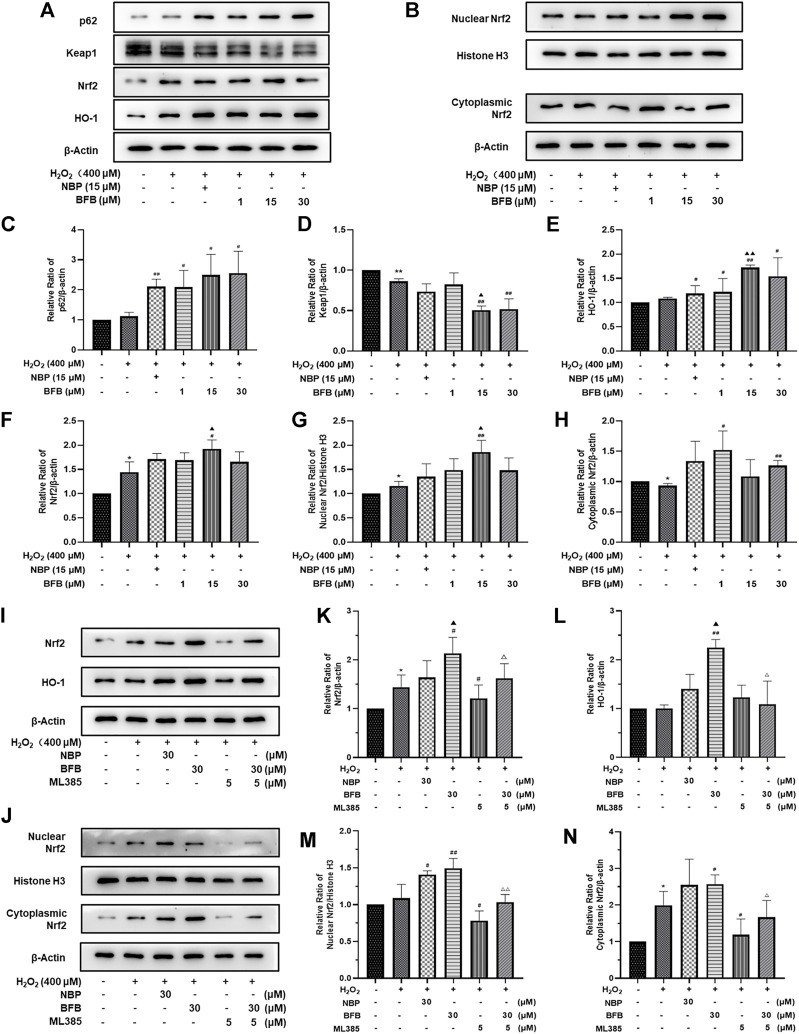
BFB’s role in cellular P62-Keap1-Nrf2 pathway-related factors protein expression level. 400 μM H_2_O_2_ treatment in PC12 cells with/without BFB (1, 5, 10 μm) or NBP (15 μm) for a 3-h period **(A)** p62, keap1, HO-1 and total Nrf2 protein levels were examined by WB **(B)** Nuclear and cytoplasmic Nrf2 protein fractions were analyzed through WB **(C–H)** Quantification of relative band intensities of p62, keap1, total Nrf2, HO-1, nuclear Nrf2 and cytoplasmic Nrf2, and β-actin or Histone H3 served as loading control. Nrf2 suppression mitigated BFB’s inhibition on oxidative stress. 5 μm ML385 was added to pretreat PC12 cells for a 0.5-h period, followed by stimulation using H_2_O_2_ (400 μm) with/without BFB (30 μm) or NBP (30 μm) **(I)** Total Nrf2 and HO-1 protein expression determined through WB assay **(J)** Nuclear and cytoplasmic Nrf2 protein determined by Western Blot **(K–N)** Quantification of relative band intensities of total Nrf2, HO-1, nuclear Nrf2 and cytoplasmic Nrf2, with β-actin or Histone H3 being the endogenous reference. The values were represented by mean ± SD from 3 separate assays. *p < 0.05, **p < 0.01, versus Control, ^#^ *p* < 0.05, ^##^ *p* < 0.01, versus H_2_O_2_ group. ^▲^ *p* < 0.05, ^▲▲^ *p* < 0.01, versus NBP group. ^△^ *p* < 0.05, ^△△^ *p* < 0.01, versus ML385 group.

For verifying BFB’s protection mechanism within H_2_O_2_-induced PC12 cells, this work analyzed the effect of BFB on Nrf2 pathway within H_2_O_2_-mediated PC12 cells injury through RT-PCR and WB assays. The p62, Nrf2, HO-1, CAT, and SOD mRNA expression increased, whereas Keap1 mRNA level decreased upon H_2_O_2_ treatment, meanwhile administration with BFB at 1, 15, 30 µm obviously promoted p62, HO-1, Nrf2, CAT, and SOD gene levels, whereas reduced Keap1 level dose-dependently (Figures 4A–F). According to WB assay, BFB exposure markedly up-regulated P62, Nrf2, HO-1 protein levels compared with H_2_O_2_ group. Moreover, administering BFB apparently increased the nuclear, cytoplasmic and total Nrf2 protein fractions compared with H_2_O_2_ group (Figures 5A–H).

The results showed that BFB could markedly elevate Nrf2 mRNA and protein levels. Thereafter, this work analyzed the role of Nrf2 suppression in blocking BFB’s impact on oxidative stress *via* ML385, the new and specific inhibitor of Nrf2. According to Figures 4G–J, H_2_O_2_-mediated up-regulation of Nrf2, HO-1, CAT, SOD mRNA levels was not reduced *via* BFB within ML385-pretreated PC12 cells. Thereafter, WB results demonstrated that ML385 exposure remarkably decreased BFB-induced Nrf2 and target activation. Therefore, Nrf2 signaling pathway was related to BFB’s protection (Figure 5I–N).

### 3.5 BFB Promoted Cellular Nrf2 Expression and Elevated Nuclear Translocation

**FIGURE 6:**
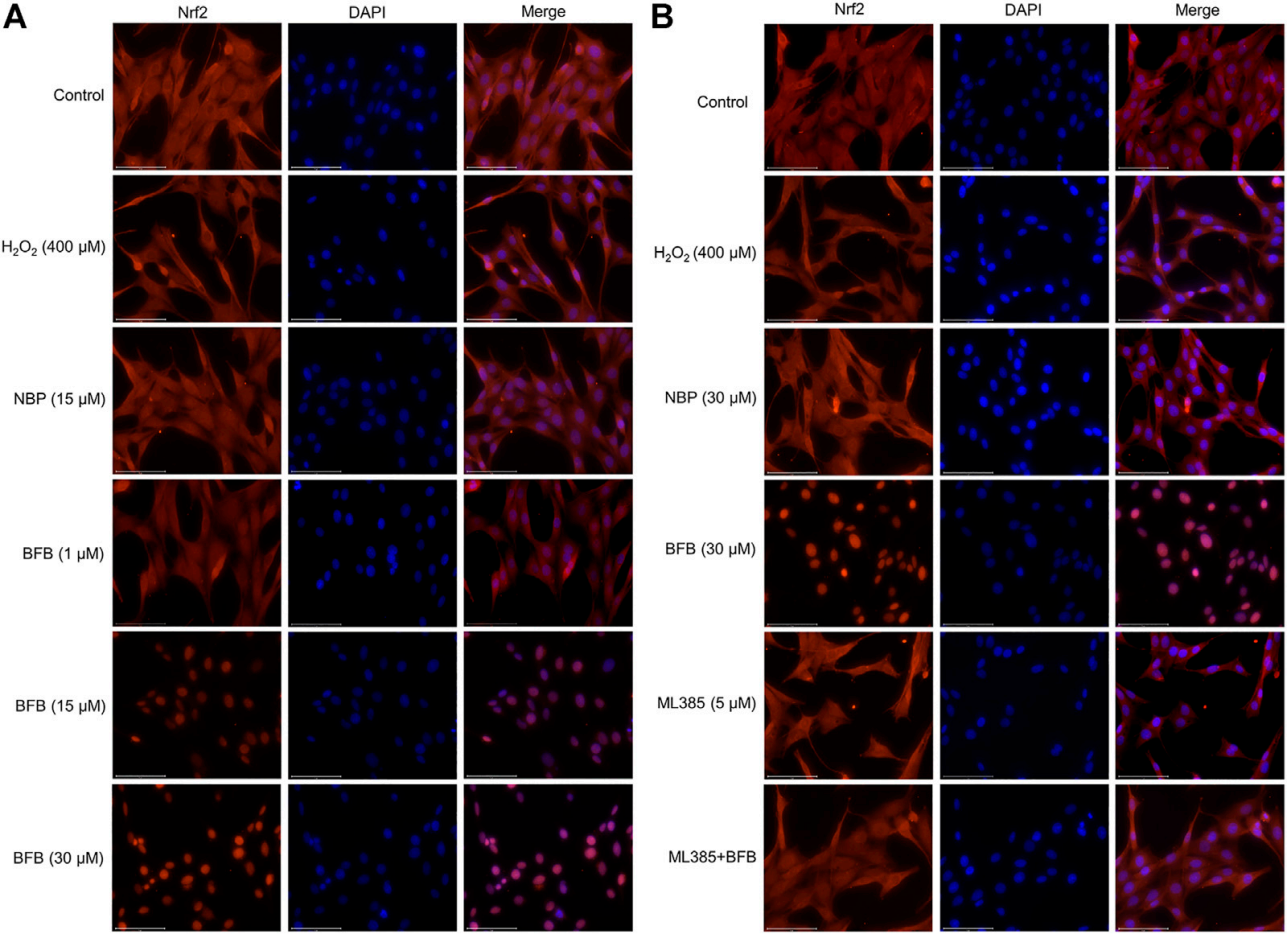
BFB promoted Nrf2 nuclear translocation. Nrf2 protein localization was detected *via* immunofluorescence staining **(A)** Representative immunofluorescence images showing BFB promoted distribution of Nrf2 in nuclear fraction **(B)** Nrf2 inhibition blocked cellular Nrf2 distribution induced by BFB in nuclear fraction.

Immunofluorescence staining was conducted to observe Nrf2 protein level. BFB pretreatment enhanced the fluorescence intensity, in particular for nucleus (Figure 6A). Therefore, BFB markedly enhanced and activated Nrf2s. ML385 pretreatment abolished BFB’s role in the expression of the Nrf2 level (Figure 6B).

### 3.6 Nrf2 Suppression Mitigated BFB’s Inhibition on H_2_O_2_-Mediated Oxidative Stress

**FIGURE 7:**
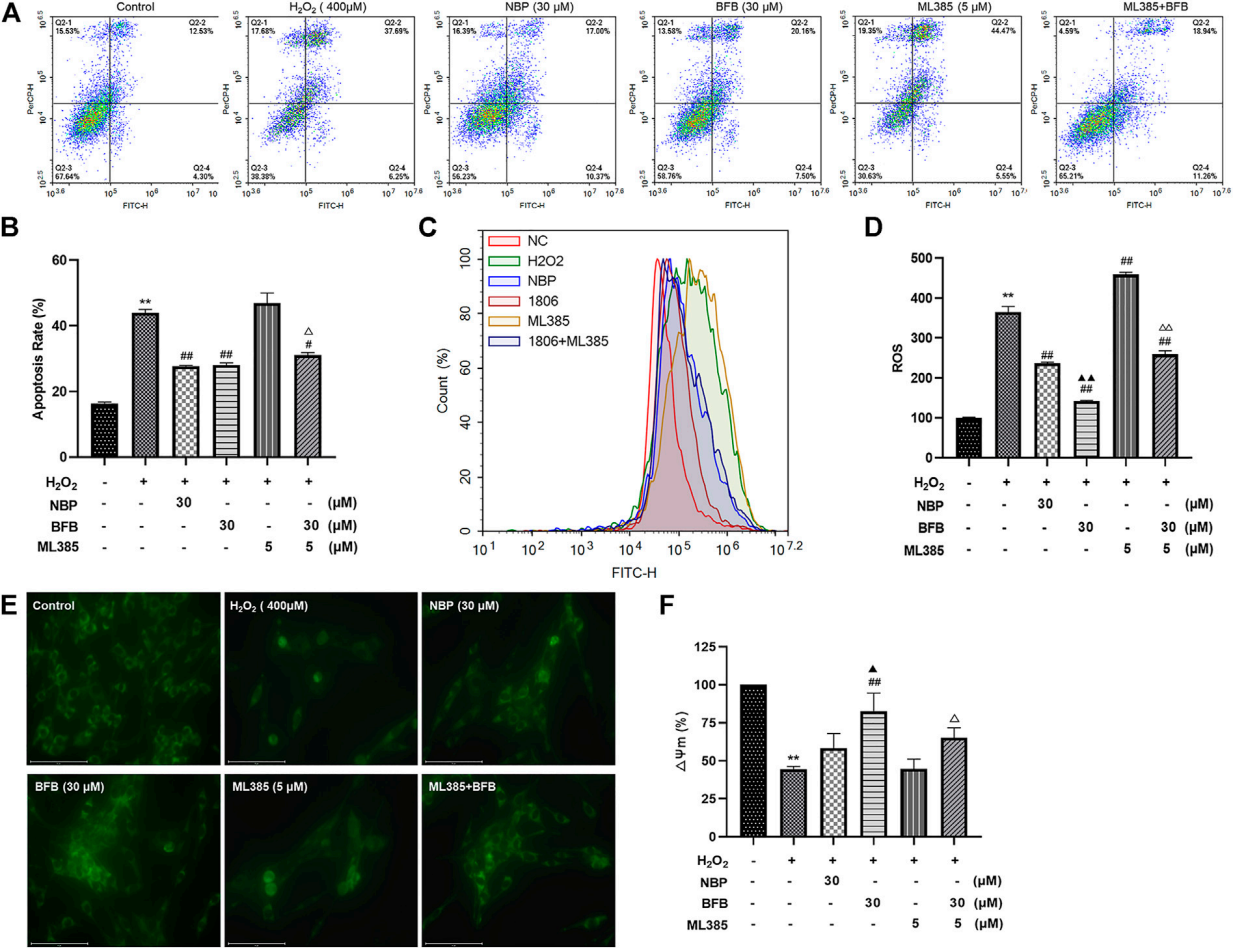
Nrf2 suppression mitigated BFB’s inhibition on oxidative stress. 5 μm ML385 pretreatment of PC12 cells for a 0.5-h period, followed by H_2_O_2_ (400 μm) stimulation with/without BFB (30 μm) or NBP (30 μm) **(A,B)** FCM on apoptotic rate using Annexin V/PI staining **(C,D)** FCM on ROS production using DCFH-DA **(E)** Typical images presenting mitochondrial staining under diverse conditions by using fluorescence microscopy (scale bar = 100 µm) **(F)** Bar graph presenting fluorescence intensity, which indicated MMP, by using fluorescence microplate reader. Data were represented by mean ± SD from 3 separate assays. *p < 0.05, **p < 0.01, versus Control, ^#^ *p* < 0.05, ^##^ *p* < 0.01, versus H_2_O_2_ group. ^▲^ *p* < 0.05, ^▲▲^ *p* < 0.01, versus NBP group. ^△^ *p* < 0.05, ^△△^ *p* < 0.01, versus ML385 group.

As shown in Figures 7A–F, CA did not block the H_2_O_2_-mediated increased apoptosis rate as well as ROS contents and decreased MMP expression within ML385-treated PC12 cells. Based on these results, Nrf2 was related to BFB’s cytoprotection.

### 3.7 BFB Protects Against H_2_O_2_-Induced Oxidative Stress by Increasing Expression of Autophagy-Related Proteins

**FIGURE 8:**
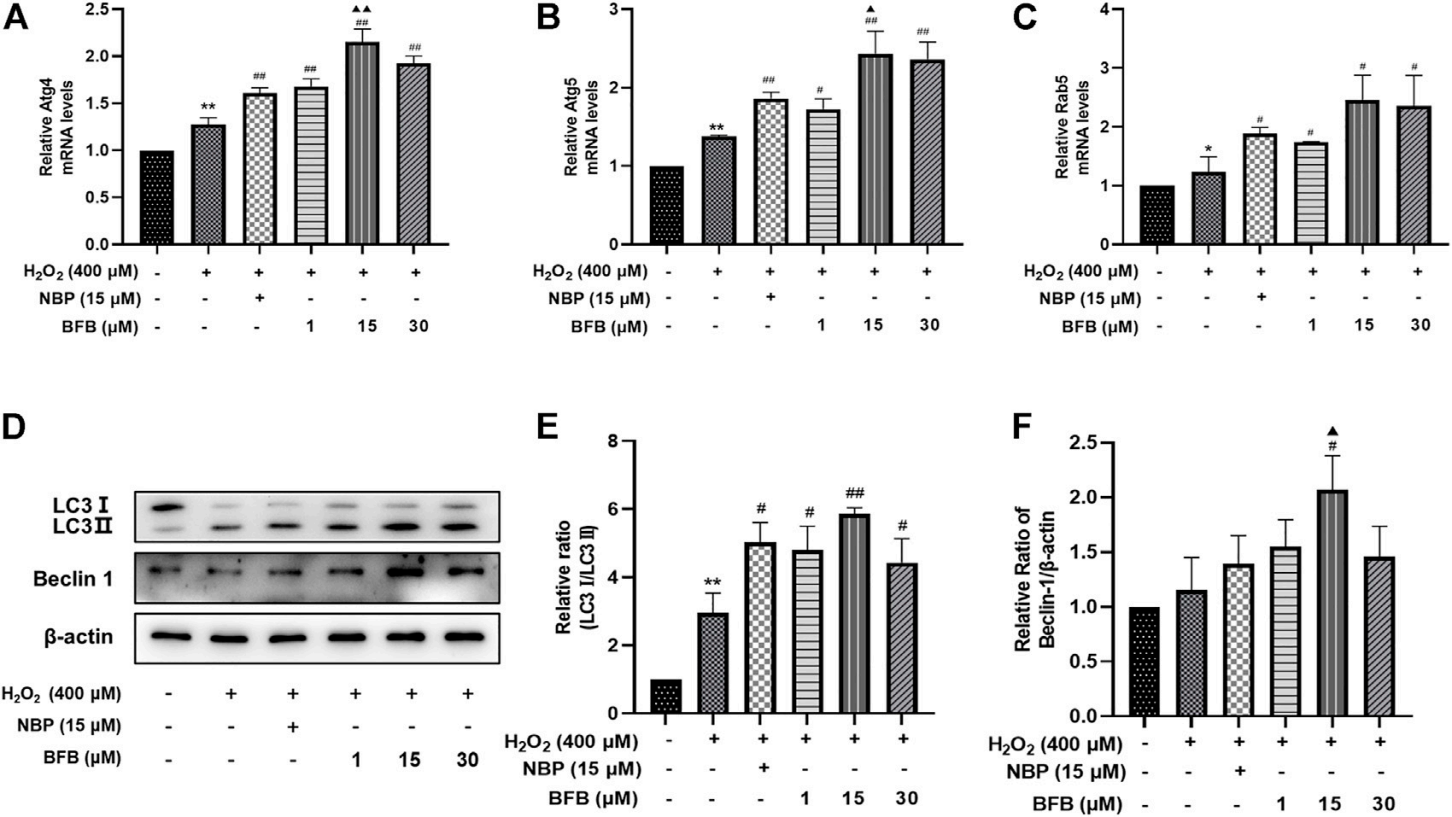
BFB inhibits oxidative stress by initiating and inducing autophagy of H_2_O_2_-mediated PC12 cells **(A–C)** Autophagy-associated gene levels (Atg 4, Atg 5, Rab 5) were tested respectively *via* RT-qPCR **(D,E)** The autophagy-associated proteins expression (LC3, Beclin-1) was measured *via* WB. Data were represented by mean ± SD of 3 separate assays. *p < 0.05, **p < 0.01, versus Control, ^#^ *p* < 0.05, ^##^ *p* < 0.01, versus H_2_O_2_ group. ^▲^ *p* < 0.05, ^▲▲^ *p* < 0.01, versus NBP group.

As shown in Figures 8A–C, RT-qPCR analysis revealed the increased Ag4, Atg5, and Rab5 mRNA levels upon H_2_O_2_ treatment, meanwhile administration with BFB at 1, 15, 30 µm obviously increased the expression of Ag4, Atg5, and Rab5 genes. Thereafter, as revealed by WB assay, BFB exposure dramatically increased LC3Ⅱ and Beclin-1 proteins expression, especially increased the ratio of LC3Ⅱ/LC3Ⅰ, as compared to H_2_O_2_ group (Figures 8D–F).

## 4 Discussion

The present study found that BFB reversed H_2_O_2_-induced lipid peroxidation, generation of ROS, reduction of MMP and massive apoptosis in PC12 cells, indicating that the protective effect of BFB on the H_2_O_2_-induced cytotoxicity would be attributed to the inhibition of oxidative stress injury. In addition, the pro-survival role of BFB was also characterized by its activation on Nrf2 signaling pathway, initiation and induction of autophagy.

Oxidative stress is responsible for the cerebral ischemia-reperfusion injury that leads to apoptosis, autophagy, and necrosis of the brain cells (Deng et al., 2016). Excess ROS produced due to oxidative stress reacts rapidly with several sites on the cytoplasmic membrane, resulting in membrane protein oxidation and lipid peroxidation (Kahles and Brandes, 2012; Zhou et al., 2016). Toxic products of lipid peroxidation, such as malondialdehyde (MDA), have longer half-lives than free radicals (Mao et al., 1999). These products migrate to different parts of the neuron, causing multiple deleterious changes in cellular function, and eventually neuronal death (Marnett, 1999). Such markers indicate a free radical attack to the phospholipids. This is accompanied with the release of lactate dehydrogenase (LDH), a key anaerobic enzyme prevalent in the cytoplasm (Niemann et al., 2017); its release directly reflects the state of the cell membrane, direction of oxygen action, and metabolic rate. When the body is exposed to oxidative damage, various antioxidants and enzymes are produced to counteract the damage (Niemann et al., 2017). GSH is a non-enzymatic antioxidant that visibly reflects the body’s antioxidant capacity (Poprac et al., 2017). This study demonstrated that BFB significantly inhibits the oxidative stress cell death of PC12 caused by H_2_O_2_ treatment, ROS aggregation, LDH and MDA levels, and enhanced GSH level.

Mitochondrial dysfunction is not only a hallmark of ischemic stroke but also involved in the pathological processes of ischemia and reperfusion (Vosler et al., 2009). As a source of cellular power, mitochondria play an essential role in cellular energy homeostasis and neuronal death, including apoptosis and autophagy, after ischemic stroke. Therefore, mitochondria are necessary for promoting neurological survival and improvement after ischemic stroke and are important drug targets in the treatment of stroke (Huang et al., 2016). Mitochondrial membrane potential (MMP) is the electrochemical potential formed between two sides of the inner mitochondrial membrane during respiratory oxidation (Lemasters et al., 2002; Hofmeijer and Van Putten, 2012). Normal MMP can maintain the function of mitochondrial ATP production and oxidative phosphorylation; however, several studies have found that MMP decreases along with apoptosis (Lemasters et al., 2002). The proteins involved in apoptosis, such as caspase-3, Bax, Bcl-10, and AIF, are synergistically upregulated with signaling proteins that regulate different apoptotic pathways. Various anti-apoptotic proteins, such as Bcl-2, p63 are also upregulated (Engel et al., 2011). Therefore, the interplay between anti-apoptotic and pro-apoptotic proteins is crucial in determining the fate of neurons in ischemic stroke (Inta et al., 2006). This study showed that H_2_O_2_ significantly reduced MMP, and mitochondrial membranes showing ruptured and loosely or irregularly arranged cristae; and increased the rate of apoptosis, and the ratio of Bax/Bcl-2 and cleaved caspase-3/caspase-3. BFB enhanced cellular MMP, improved mitochondrial morphology, and reversed the above data.

Studies have identified that Nrf2 plays a crucial role in maintaining cellular redox homeostasis and regulating inflammatory responses in the mechanism of an oxidative stress injury in neurodegenerative diseases (Cheng et al., 2021). Nrf2 and its endogenous inhibitor Keap1 are a prevalent and evolutionarily conserved intracellular defense mechanism (Eftekharzadeh et al., 2010). Under normal conditions, Nrf2 is isolated by cytoplasmic Keap1 and degraded by targeted proteases. In contrast, when the organism undergoes peroxidative damage, Nrf2 detaches from Keap1 and translocates to the nucleus, where it binds to a small Maf protein as a heterodimer (Zhang, 2006; Eftekharzadeh et al., 2010). The heterodimer can recognize the ARE sequence, an enhancer sequence present in the regulatory region of Nrf2 target genes, essential for the recruitment of key transcription factors and the activation of antioxidant enzymes. Therefore, small molecule compounds that can activate the Nrf2 pathway are promising neuroprotective agents (Esposito et al., 2017; Fão et al., 2019). Our experimental results showed that BFB differentially enhanced the expression of P62-Keap1-Nrf2 pathway-related factors, promoted the translocation of Nrf2 to the nucleus, and activated the antioxidant enzymes HO-1, CAT, and SOD. ML385 blocked the effect of BFB on the expression of Nrf2 pathway-related factors.

In addition, compared with the BFB, the upstream regulator of the Nrf2 pathway, p62 (p62/SQSTM1), is also important in mediating autophagy. LC3 binds phosphatidylethanolamine during autophagic membrane prolongation to form LC3-Ⅱ, which is a necessary process for in autophagy, and p62 is a selective substrate for autophagic degradation. Therefore, the conversion of LC3 from LC3-Ⅰ to LC3-Ⅱ as well as the p62 content can be used as markers of autophagy (Noda and Inagaki, 2015; Lamark et al., 2017). In the absence of nutrients or growth factors, autophagy is considered non-selective and mainly increases the degradation of any cytoplasmic proteins and other macromolecules to provide essential nutrients (Corti et al., 2020). In contrast, autophagy is considered selective under stress conditions, purposefully recognizing substrates and binding them tightly to the emerging autophagosomal membrane (Menzies et al., 2015). The target of rapamycin protein (mTOR), an important regulator of autophagy, is inhibited by its complex mTORC1 under normal conditions. Beclin-1 forms a complex with Beclin-2 on the endoplasmic reticulum (ER), while mTORC1 inactivation mediates autophagy during the onset of oxidative stress in the organism (Noda, 2017). This process is accompanied by the activation of the ULK1 complex, Atg family, Beclin-1, and PI3K complex, promoting the formation of autophagic precursor membranes (Neufeld, 2020). The Atg family plays a key role in the extension of the autophagosomal membrane with LC3 and P62 to promote the generation of autophagosomes, mediating the eventual binding of autophagosomes to lysosomes for autolysis, degradation of substrates, and provision of energy (Noda and Inagaki, 2015; Nakatogawa, 2020). The results showed that BFB pretreatment significantly increased Atg4, Atg5, Rab5, LC3 protein, LC3Ⅱ/LC3Ⅰ, and Beclin-1 protein expressions than the H_2_O_2_ group exerting its protective effect on H_2_O_2_-induced oxidative stress injury in PC12 cells.

Some researches reported that NBP can elevate mitochondrial function, balance the ratio of pro-apoptosis protein and anti-apoptosis protein *via* the activation of Nrf-2signaling pathway. We also demonstrated above results in this study. BFB is a derivative of NBP, Therefore, the protective mechanism of BFB is similar to NBP. However,. Our previous study showed that BFB is more active than NBP and its LD_50_ is more than that of NBP’s on mouse acute toxicity (data not shown).

In conclusion, there are few researches concentrate upon the activation of autophagy and Nrf2 signaling pathway simultaneously by p62-Keap1-Nrf2 feedback axis to treat cerebral ischemic stroke, and further investigation and study are needed until now. The strategy of treating cerebral ischemic stroke by targeting the p62-Keap1-Nrf2 feedback axis supply for the novel perspectives for the development of new drugs in the future. The present study demonstrated that BFB inhibits oxidative stress by activating Nrf2 to initiate and induce autophagy, consequently improving cell survival. BFB maybe a candidate of the agent for cerebral ischemia.

## Data Availability

The original contributions presented in the study are included in the article/Supplementary Materials, further inquiries can be directed to the corresponding author.
